# Cancer-derived exosomal miR-138-5p modulates polarization of tumor-associated macrophages through inhibition of KDM6B

**DOI:** 10.7150/thno.51864

**Published:** 2021-05-03

**Authors:** Jing Xun, Lingfang Du, Ruifang Gao, Long Shen, Dekun Wang, Lichun Kang, Chuan'ai Chen, Zhujun Zhang, Yuying Zhang, Shijing Yue, Shuxin Feng, Rong Xiang, Xue Mi, Xiaoyue Tan

**Affiliations:** 1 School of Medicine, Nankai University, Tianjin 300071, China.; 2 Tianjin Nankai Hospital, Tianjin, 300100, China.; 3 Department of Orthopedics, Tianjin First Central Hospital, Tianjin, 300071, China.

**Keywords:** tumor-associated macrophages, lysine demethylase 6B (KDM6B), microRNA-138-5p, exosomes, macrophage polarization

## Abstract

**Rationale:** Differential activation of macrophages correlates closely with tumor progression, and the epigenetic factor lysine demethylase 6B (KDM6B, previously named JMJD3) mediates the regulation of macrophage polarization through an unknown mechanism.

**Methods:** We developed a suspension coculture system comprising breast cancer cells and macrophages and used RT-qPCR and western blotting to measure *KDM6B* expression. Bioinformatics and luciferase reporter assays were used to identify candidate microRNAs of cancer cells responsible for the downregulation of KDM6B. To determine if exosomes mediated the transfer of miR-138-5p between cancer cells to macrophages, we treated macrophages with exosomes collected from the conditioned medium of cancer cells. The effects of exosomal miR-138-5p on macrophage polarization were measured using RT-qPCR, flow cytometry, and chromatin immunoprecipitation assays. We employed a mouse model of breast cancer, metastatic to the lung, to evaluate the effects on tumor metastasis of macrophages treated with miR-138-5p-enriched exosomes. To develop a diagnostic evaluation index, the levels of exosomal miR-138-5p in samples from patients with breast cancer were compared to those of controls.

**Results:** Coculture of breast cancer cells led to downregulation of *KDM6B* expression in macrophages. Cancer cell-derived exosomal miR-138-5p inhibited M1 polarization and promoted M2 polarization through inhibition of *KDM6B* expression in macrophages. Macrophages treated with exosomal miR-138-5p promoted lung metastasis, and the level of circulating exosomal miR-138-5p positively correlated with the progression of breast cancer.

**Conclusion:** Our data suggest that miR-138-5p was delivered from breast cancer cells to tumor-associated macrophages via exosomes to downregulate *KDM6B* expression, inhibit M1 polarization, and stimulate M2 polarization. Therefore, exosomal miR-138-5p represents a promising prognostic marker and target for the treatment of breast cancer.

## Introduction

Tumor-associated macrophages (TAMs), which are critical components of the tumor microenvironment, are widely involved in the regulation of tumor progression 1. Macrophages uniquely change their phenotype, and thus are accordingly classified into the major subtypes as follows: classically activated (M1) and alternatively activated (M2) 2. Diverse polarized macrophage populations are associated with distinct tumor fates, and M2-like macrophages correlate with poor prognosis of patients with cancer 3, 4. Such plasticity, as well as their key role in tumor progression, strongly indicates that the components of the mechanism responsible for reprogramming macrophage will serve as targets of cancer therapy 5, 6.

Transcription factor activity, epigenetic modifications, and the products of metabolic pathways modulate the differential activation of macrophages 6, 7. For example, epigenetic remodeling mediated by histone-modifying enzymes is associated with modulation of TAM phenotypes. Lysine demethylase 3B (KDM3B, also named JMJD1A) reduces the number of TAMs and their proangiogenic activity 8. Substrates of lysine demethylase 6B (KDM6B, previously named JMJD3) include tri- and dilysine residue 27 of histone H3 (H3K27me3/2), which is involved in activating gene transcription required to regulate macrophage activation 9. Moreover, LPS, IL-4, or helminth infection induces KDM6B, leading to the modulation of macrophage activation 10-12. However, the regulations of KDM6B expression, as well as the mechanism underlying the effect of KDM6B on the polarization of TAMs are still far from clear.

miRNAs are endogenous small noncoding RNAs comprising 20-25 nucleotides that function as posttranscriptional repressors of gene expression by targeting the 3'-untranslated regions (UTRs) of target mRNAs. Increasing evidence reveals that miRNAs are required for the regulation of the differential activation of macrophages during tumor progression 13, 14. Moreover, miRNAs are generally involved in intercellular cross-talk. For example, tumor cells secret different miRNAs that enter neighboring cells or circulate, contributing to tumor progression 15-19. Here we show that miRNA-138-5p derived from tumor cells influences the expression and activity of the critical epigenetic regulator KDM6B in macrophages and thus influences macrophage polarization.

The instability of miRNAs requires a specific cargo to deliver them to target cells. For example, exosomes, which are small vesicles enclosed in a lipid-bilayer membrane (30-100 nm diameter), represent an important paradigm of intercellular communication 20, 21. Tumor-derived exosomes deliver diverse molecules such as proteins 22, lipids, and nucleic acids that contribute to the modification of the tumor microenvironment and the establishment of a pro-metastatic niche 23-26. Moreover, evidence indicates that tumor-derived exosomes deliver miRNAs to TAMs, leading to changes in metabolism and inflammation 15, 27, 28. Here, we show that the delivery of miR-138-5p to macrophages is mediated by exosomes and propose that exosomal miR-138-5p will serve as a prognostic biomarker for tumor progression.

Here we present evidence for the downregulation of *KDM6B* expression in macrophages cocultured with miR-138-5p-enriching breast cancer cells. We further show that miR-138-5p inhibited *KDM6B* expression and that cancer cell-derived exosomes delivered miR-138-5p to macrophages, leading to the inhibition of the M1 phenotype, while activating M2 polarization. Moreover, we show that administration of macrophages treated with exosomal miR-138-5p promoted lung metastasis in a mouse model of metastatic breast cancer and that the circulating level of exosomal miR-138-5p positively correlated with cancer progression in patients with breast cancer. These findings strongly suggest that exosomal miR-138-5p might serve as a prognostic marker for tumor progression as well as a target of therapy.

## Results

### The expression of *KDM6B* by macrophages is inhibited by miR-138-5p

Here, we first examined the effect of MB-MDA-231 breast cancer cells on *KDM6B* expression in the macrophage-like cell line THP-1. After 48 h of suspension coculture with MDA-MB-231, KDM6B levels significantly decreased in THP-1 cells, although a slight increase was detected after 12 h (Figure S1A-B). Conditioned medium (CM) derived from MDA-MB-231 suppressed *KDM6B* expression in THP-1 cells (Figure S1C), suggesting that a factor secreted from the breast cancer cells was responsible for this effect.

**Figure 1:**
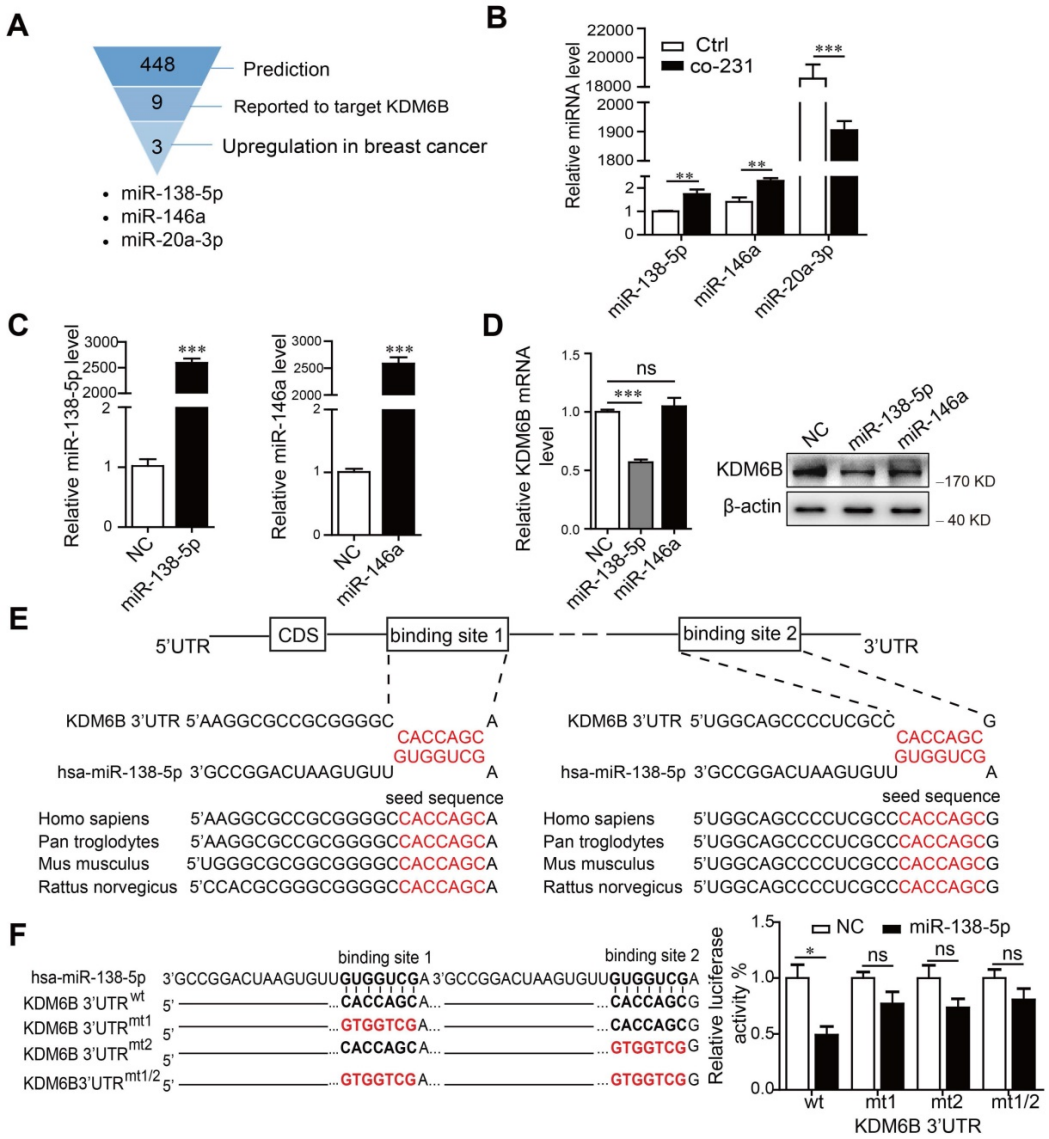
** miR-138-5p inhibits the expression of KDM6B in macrophages.** THP-1 cells were cocultured with MDA-MB-231 cells for 48 h. (A) Schematic of screening for candidate miRNAs. (B) RT-qPCR analysis of miR-138-5p, miR-146a, and miR-20a-3p expression in THP-1 cells. U6 was used as an internal control. (C) RT-qPCR analysis of miR-138-5p and miR-146a expression in THP-1 cells transfected with negative control (NC), miR-138-5p or miR-146a mimics for 48 h. (D) RT-qPCR and western blot analyses of *KDM6B* expression in THP-1 cells transfected with NC, miR-138-5p or miR-146a mimics. (E) Predicted miR-138-5p binding sites in the 3´-UTR of *KDM6B*, and the seed sequences of miR-138-5p of different species. (F) The mutant construct with miR-138-5p binding sites in the 3´-UTR of *KDM6B* (left). The pmirGLO reporter harboring the 3´-UTR of KDM6B with wild-type (wt) or mutated (mt) miR-138-5p binding sites were used to cotransfected THP-1 cells with miR-138-5p mimics or NC. Luciferase activity was analyzed 40 h after transfection (right). The results are shown as the mean ± SEM of three independent experiments. *P < 0.05; **P < 0.01; ***P < 0.001.

miRNAs are involved in signal transduction among distinct cell types that populate the tumor microenvironment. We therefore attempted to determine if miRNAs contributed to the regulation of *KDM6B* expression. For this purpose, we conducted bioinformatics analysis and a review of the literature to identify miRNAs targeting the 3'-untranslated regions (3'-UTR) of *KDM6B*. We identified miR-138-5p, miR-146a, and miR-20a-3p as candidate miRNAs mediating the inhibition by cancer cells of the expression of *KDM6B* in macrophages (Figure 1A). When we analyzed the changes of miRNA levels in THP-1 cells after coculture with MDA-MB-231 cells, we found that those of miR-138-5p and miR-146a significantly increased, while those of miR-20a-3p decreased (Figure 1B).

Considering that miRNA levels correlate negatively with those of target genes in general, we next confirmed whether miR-138-5p or miR-146a inhibited *KDM6B* expression. We found that ectopic expression of miR-138-5p, but not miR-146a, inhibited *KDM6B* expression in THP-1 cells (Figure 1C-D). Sequence analysis showed that miR-138-5p contains two potential target sites in the 3-'UTR of *KDM6B* mRNA. Moreover, the target-binding sequences of miR-138-5p in the 3-'UTR of *KDM6B* mRNA were highly conserved among numerous, diverse species (Figure 1E).

Luciferase reporter assays showed that overexpression of miR-138-5p suppressed the luciferase activity driven by the 3'-UTR of* KDM6B* mRNA, but not that of a mutated cognate 3'-UTR (Figure 1F). These data suggest that miR-138-5p suppressed *KDM6B* expression via binding to its 3'-UTR.

### miR-138-5p mediates cancer cell-induced inhibition of *KDM6B* expression in macrophages

**Figure 2:**
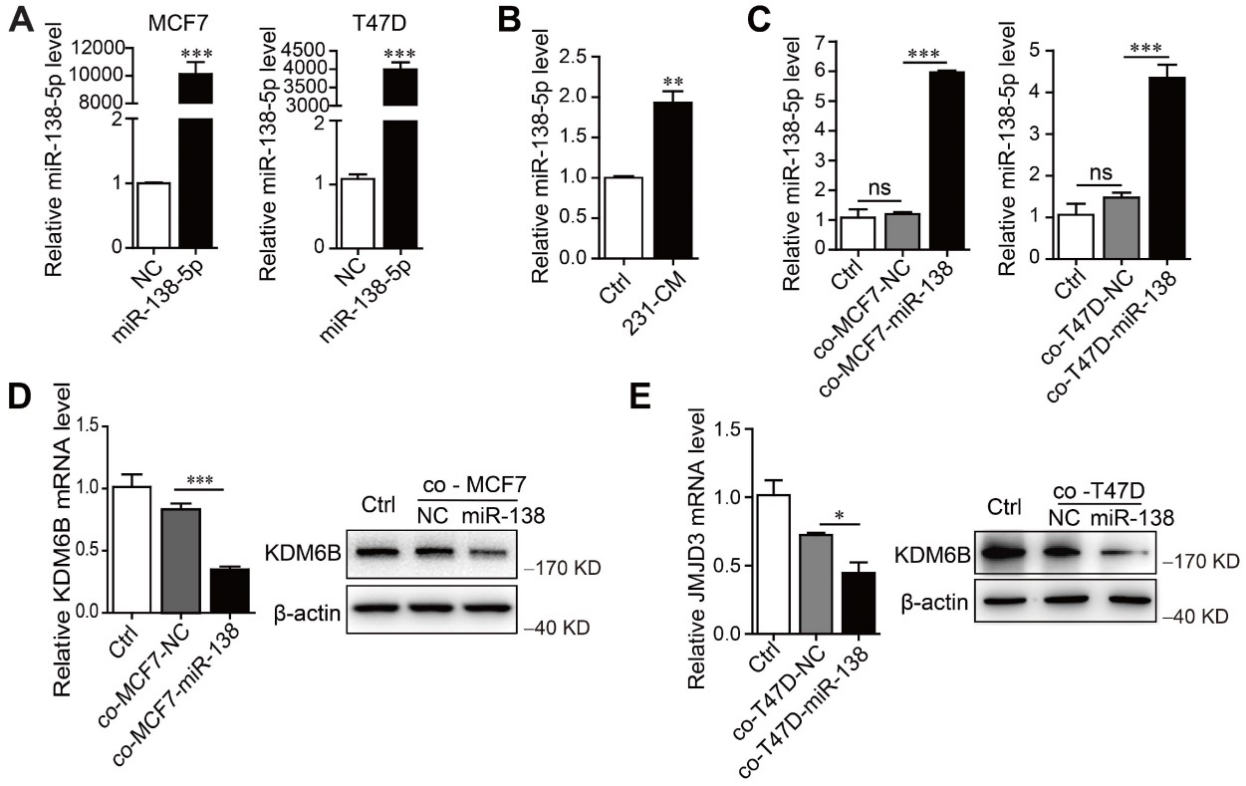
** miR-138-5p mediates cancer cell-induced inhibition of KDM6B expression in macrophages.** THP-1 cells were cocultured with MDA-MB-231 cells for 48 h. (A) RT-qPCR analysis of miR-138-5p expression in MCF7 and T47D cells transfected with NC or miR-138-5p mimics. (B) RT-qPCR analysis of miR-138-5p expression in THP-1 cells treated with conditioned medium (CM) of cultures of MDA-MB-231 cells for 48 h. THP-1 cells were cocultured for 48 h with MCF7 or T47D cells transfected NC or miR-138-5p mimics. (C) RT-qPCR analysis of levels of miR-138-5p in THP-1 cells. (D, E) The analysis of *KDM6B* mRNA and protein levels in THP-1 cells. The results are shown as the mean ± SEM of three independent experiments. *P < 0.05; **P < 0.01; ***P < 0.001.

To determine whether miR-138-5p mediated the inhibitory effect of MDA-MB-231 cells on *KDM6B* expression in macrophages, we first measured miR-138-5p levels in phenotypically distinct breast cancer cell lines, as well as in the nontumorigenic cell line MCF10A. According to their levels (low or high) of miR-138-5p, we identified MCF7 and T47D cells as miR-138-5p low and MDA-MB-231 as miR-138-5p high (Figure S2A). When we transfected MCF7 and T47D cells with miR-138-5p mimics and ectopically overexpressed miR-138-5p (Figure 2A), we found that treatment with the CM of MDA-MB-231 cells or coculture with MCF7 and T47D cells ectopically overexpressing miR-138-5p increased the levels of miR-138-5p expressed by THP-1 cells (Figure 2B-C). Moreover, coculture with ectopic MCF7 and T47D cells ectopically overexpressing miR-138-5p inhibited *KDM6B* mRNA and protein expression in THP-1 cells (Figure 2D-E). We concluded therefore that miR-138-5p mediated the inhibition of *KDM6B* expression induced by coculture of THP-1 cells with cancer cells.

### Exosomal miR-138-5p produced by cancer cells downregulates *KDM6B* expression in macrophages

**Figure 3:**
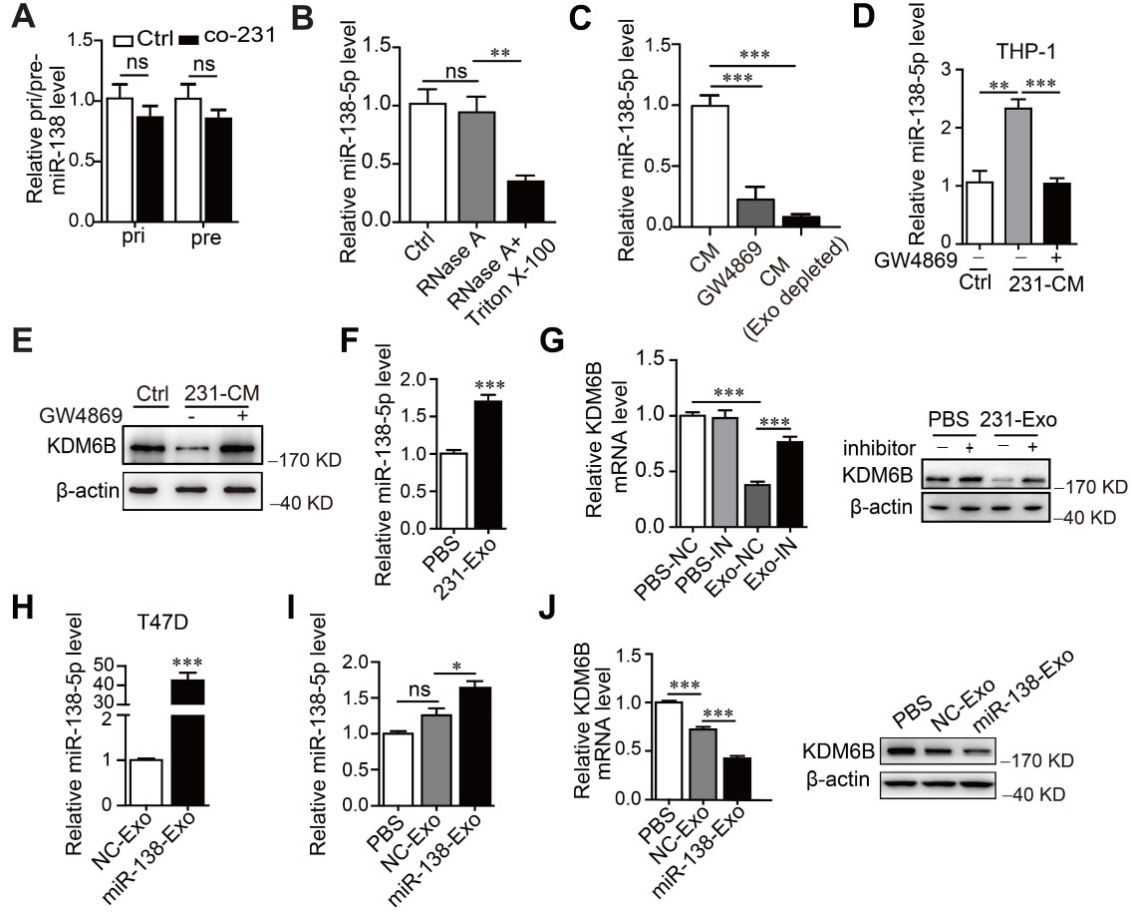
** Cancer cell-derived exosomal miR-138-5p downregulates KDM6B expression.** THP-1 cells were cocultured with MDA-MB-231 cells for 48 h. **(A)** RT-qPCR analysis of pri-miR-138 and pre-miR-138 expression in THP-1 cells. (B) RT-qPCR analysis of the expression levels of miR-138-5p in the CM of MDA-MB-231 cells treated with RNase A alone or in combination with Triton X-100. **(C)** RT-qPCR analysis of miR-138-5p levels in the CM of MDA-MB-231 cells depleted of exosomes using GW4869 or ultracentrifugation. CM of MDA-MB-231 cells treated with 10 μM GW4869 were collected. (D) RT-qPCR analysis of miR-138-5p expression in THP-1 cells treated with the indicated conditioned medium for 48 h. (E) Western blot analysis of KDM6B expression in THP-1 cells. (F) RT-qPCR analysis of miR-138-5p expression in THP-1 cells treated with exosomes from MDA-MB-231 cells. (G) THP-1 cells transfected with NC or an miR-138-5p inhibitor were treated with PBS or exosomes from MDA-MB-231 cells for 48 h. RT-qPCR and western blot analyses *KDM6B* expression in the indicated THP-1 cells. (H) RT-qPCR analysis of miR-138-5p levels in the exosomes derived from T47D cells transfected with NC or miR-138-5p mimics for 48 h. (I) RT-qPCR analysis of miR-138-5p expression in THP-1 cells treated with PBS or exosomes derived from T47D cells transfected with NC or miR-138-5p mimics. (J) RT-qPCR and western blot analysis of *KDM6B* expression in THP-1 cells treated with PBS or its levels in exosomes from T47D cells transfected with NC or miR-138-5p mimics. The results are shown as the mean ± SEM of three independent experiments. *P < 0.05; **P < 0.01; ***P < 0.001.

We next asked how the levels of miR-138-5p increased under the conditions described above. For this purpose, we measured the levels of primary (pri-) and precursor (pre-) miR-138 in macrophages. We found that pri- or pre-miR-138 did not increase in THP-1 cells after treatment, indicating that miR-138-5p was exogenously supplied (Figure 3A). Furthermore, the levels of miR-138-5p were unchanged when we treated the CM of MDA-MB-23 with RNase A, in contrast to treatment with RNase A plus Triton X-100 (Figure 3B). These findings supported the conclusion that extracellular miR-138-5p was encased in a membranous structure.

Further evidence implicating exosomal miR-138-5p in the regulation of *KDM6B* expression was gained through the observation that the level of miR-138-5p significantly decreased in GW4869-treated CM or exosome-depleted CM compared with untreated CM (Figure 3C). These results indicate that miR-138-5p was encapsulated within the exosomes released by breast cancer cells.

Evaluation of the exosome protein markers CD63, CD9, and Alix, as well as nanoparticle tracking analysis and transmission electron microscopy, was performed to evaluate the characteristics of the exosomes (Figure S2B-D). Furthermore, when we measured miR-138-5p levels in the exosomes isolated from diverse breast cancer cell lines, we found that they were similar to its intracellular levels (Figure S2E).

We next investigated whether exosomal miR-138-5p from breast cancer cells mediated the increase of miR-138-5p levels in THP-1 cells. We found that the inhibitor of exosomes GW4869 blocked the increase of miR-138-5p levels caused by treatment of the CM from MDA-MB-231 cells (Figure 3D). Furthermore, Cy3-labeled miR-138-5p was detected in THP-1 cells and colocalized with Dio-labeled exosomal membranes (Figure S2F). Together, our data suggest that cancer cells secreted exosomal miR-138-5p, which was transferred to macrophages.

When we measured the effect of exosomal miR-138-5p on the expression of *KDM6B* in macrophages, we found that inhibition of exosome blocked the inhibition of KDM6B induced by treating the cells with CM (Figure 3E). Treatment of MDA-MB-231-derived exosomes increased miR-138-5p levels and inhibited *KDM6B* expression in THP-1 cells, and this effect was abrogated in recipient THP-1 cells treated with an miR-138-5p inhibitor (Figure 3F-G). Transfection of T47D cells with an miR-138-5p mimic increased the levels of exosomal miR-138-5p (Figure 3H). Exosomes from T47D cells overexpressing miR-138-5p had increased miR-138-5p levels and suppressed *KDM6B* expression in THP-1 cells (Figure 3I-J). Together, these results suggest the exosomal miR-138-5p secreted by cancer cells was delivered to macrophages in which it inhibited KDM6B.

### Exosomal miR-138-5p regulates macrophage polarization by inhibiting *KDM6B* expression

**Figure 4:**
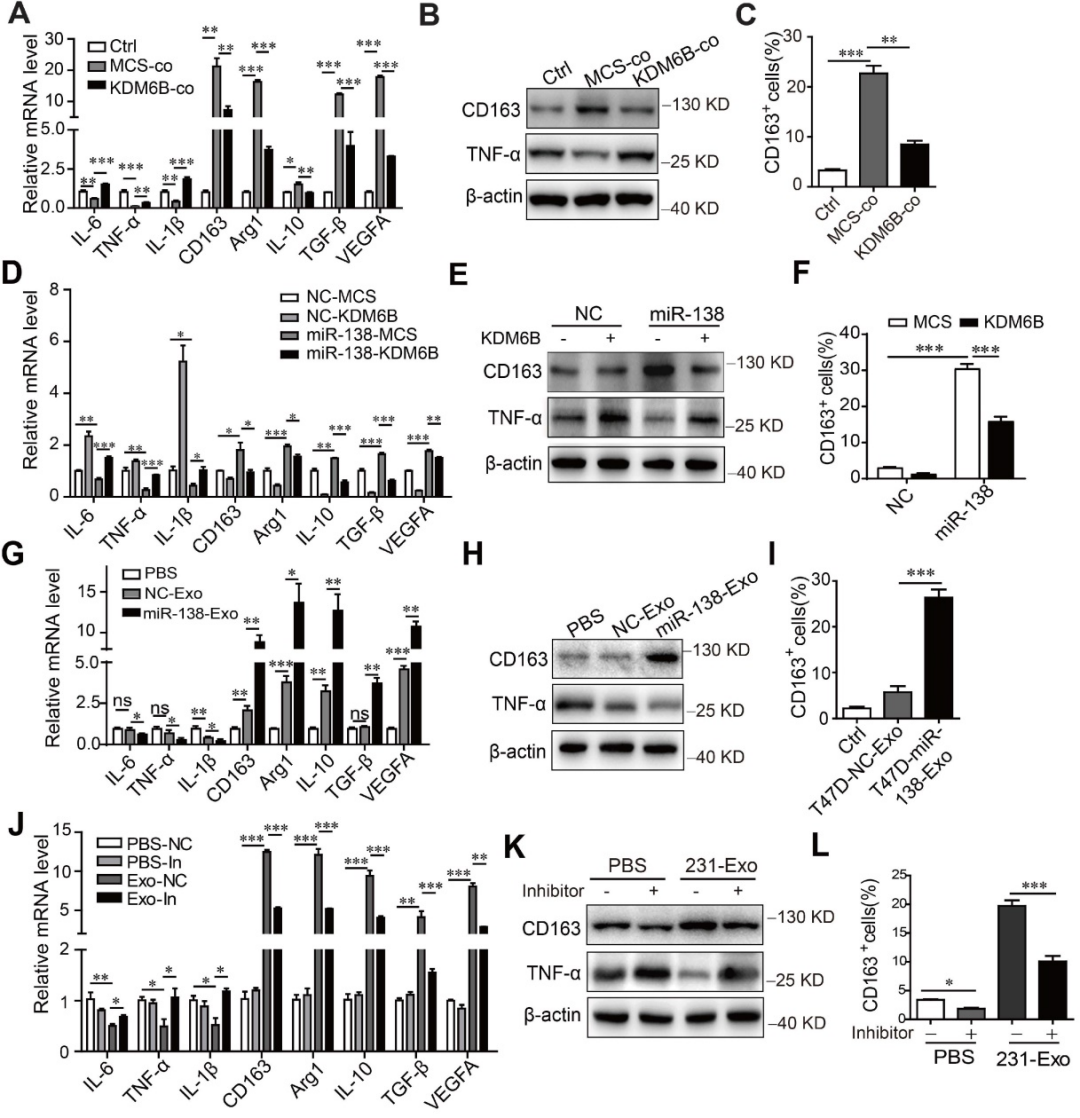
** Exosomal miR-138-5p regulates macrophage polarization via inhibiting KDM6B expression.** (A) RT-qPCR analysis of M1- and M2-associated genes, (B) western blot analysis of CD163 and TNF-α expression, and (C) Flow cytometry of CD163-positive cells in KDM6B-overexpressing THP-1 cells cocultured with MDA-MB-231 cells for 48 h. THP-1 cells overexpressing miR-138-5p or NC were transfected with the KDM6B expression plasmid or control for 48 h. (D) RT-qPCR analysis of M1- and M2-associated genes expressed in THP-1 cells. (E) Western blot analysis of CD163 and TNF-α expression in THP-1 cells. (F) Flow cytometric analysis of CD163-positive cells. (G) RT-qPCR analysis of M1- and M2-associated genes, (H) western blot analysis of CD163 and TNF-α expression, and (I) CD163-positive cells in THP-1 cells incubated with PBS or with exosomes derived from T47D cells transfected with NC or miR-138-5p mimics. (J) RT-qPCR analysis of M1- and M2-associated gene expression, (K) western blot analysis of CD163 and TNF-α expression and (L) flow cytometric analysis of CD163-positive cells in THP-1 cells. THP-1 cells transfected with NC or an miR-138-5p inhibitor were treated with PBS or exosomes from MDA-MB-231 cells for 48 h. The results are shown as the mean ± SEM of three independent experiments. *P < 0.05; **P < 0.01; ***P < 0.001.

To determine whether the inhibition of *KDM6B* expression induced by miR-138-5p influenced macrophage polarization, we determined the transcriptional profile of THP-1 cells cultured under different conditions. First, coculture with tumor cells inhibited the expression of M1-related genes encoding IL-6, TNF-α, and IL-1β and increased the expression of M2-related genes encoding CD163, Arg1, IL-10, TGF-β, and VEGFA, as well as expression of TNF-α and CD163 at protein level. Overexpression of *KDM6B* partially inhibited alternative phenotypic activation under these conditions (Figure 4A-B).

Flow cytometry showed that coculture of MDA-MB-231 cells with THP-1 cells increased the percentage of CD163-positive macrophages, which was inhibited when these cells ectopically expressed KDM6B (Figure 4C). Similarly, KDM6B overexpression inhibited M2-like polarization induced by transfection of THP-1 cells with miR-138-5p mimics (Figure 4D-F). Compared with the control, treatment of THP-1 cells with exosomes isolated from T47D cells expressing miR-138-5p decreased the M1-related phenotype. In contrast, the expression of M2 markers by macrophages increased along with the number of CD163-positive cells in macrophage cultures (Figure 4G-I).

Treatment of THP-1 cells with exosomes isolated from MDA-MB-231 cells led to M2-like polarization, which was rescued via inhibition of miR-138-5p expression (Figure 4J-L). Furthermore, transfection with miR-138-5p mimics or incubation with exosomes isolated from the CM of T47D cells overexpressing miR-138-5p increased the proliferation of THP-1 cells (Figure S3A-B).

Transwell assays revealed that the migration of MDA-MB-231 cells increased upon treatment with CM from macrophages that were incubated with exosomal miR-138-5p (Figure S3C). Together, our data indicate that exosomal miR-138-5p promoted M2-like polarization through the inhibition of *KDM6B* expression in macrophages.

### KDM6B demethylase activity promotes M1-associated gene expression

**Figure 5:**
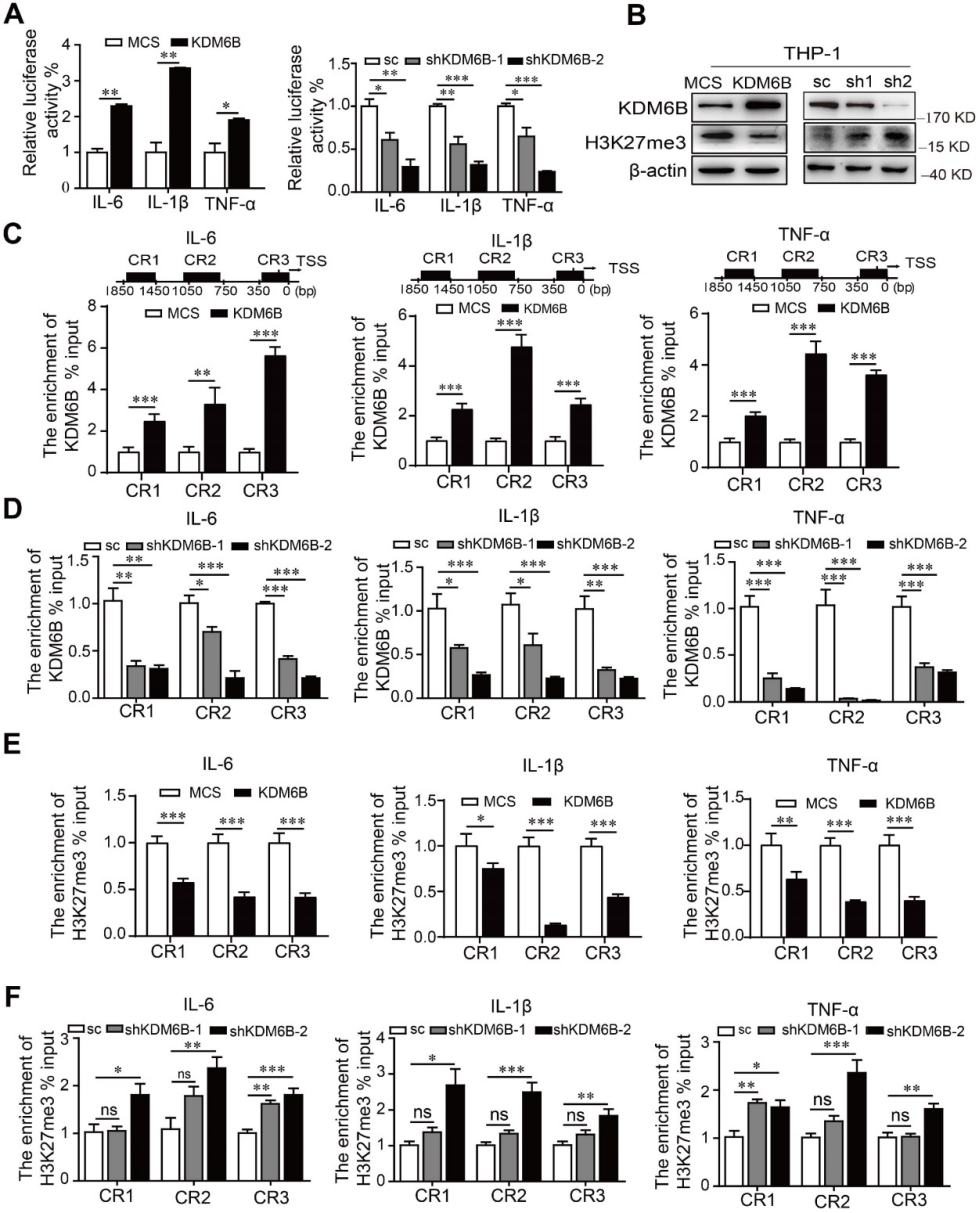
** KDM6B demethylase activity increases the expression of M1-related genes.** THP-1 cells overexpressing KDM6B were cocultured with MDA-MB-231 cells for 48 h. (A) Dual-luciferase reporter assay analysis of the promoter activities of M1-associated genes in THP-1 cells overexpressing KDM6B and controls and in THP-1-KDM6B-knockdown and scrambled-sequence control THP-1 cells. (B) Western blot analysis of KDM6B and H3K27me3 expression in THP-KDM6B and THP-shKDM6B cells, respectively. ChIP array analysis of the enrichment of KDM6B at the promoter regions of the genes encoding IL-6, TNF-α, and IL-1β in THP-1 cells overexpressing KDM6B overexpressing and control THP-1 cells (C) or THP-1-KDM6B-knockdown and scrambled-sequence control THP-1 cells (D). ChIP array analysis of the enrichment of H3K27me3 on the promoter regions of the genes encoding IL-6, TNF-α, and IL-1β in THP-1 cells overexpressing KDM6B and control THP-1 cells (E) or THP-1-KDM6B-knockdown and scrambled sequence control THP-1 cells (F) Data are shown as the mean ± SEM of three independent experiments. *P < 0.05; **P < 0.01; ***P < 0.001.

Inhibition of the histone demethylase activity of KDM6B inhibits the transcription of its target genes. Therefore, we used a dual-luciferase assay to analyze the promoter activity of M1-target genes. Overexpression of *KDM6B* specifically stimulated the promoter activities of genes encoding the proinflammatory factors IL-6, IL-1β, and TNF-α (Figure 5A). In contrast, silencing KDM6B activity led to the inhibition of their promoter activities, and *KDM6B* overexpression in THP-1 cells decreased the levels of H3K27me3 that were increased when *KDM6B* expression was inhibited (Figure 5B).

ChIP assays revealed that overexpression or knockdown of *KDM6B* in THP-1 cells significantly increased or decreased the occupancy of KDM6B at different regions of these promoters, respectively, compared with the control (Figure 5C-D). Consistent with these findings, recruitment of H3K27me3 to these promoters was decreased or increased when *KDM6B* was overexpressed or downregulated in THP-1 cells (Figure 5E-F). Therefore, downregulation of KDM6B increased the levels of H3K27me3 and the transcriptional activity of genes encoding proinflammatory factors, leading to the inhibition of M1 polarization.

### Exosomal miR-138-5p promotes the metastasis of breast cancer to the lung

**Figure 6:**
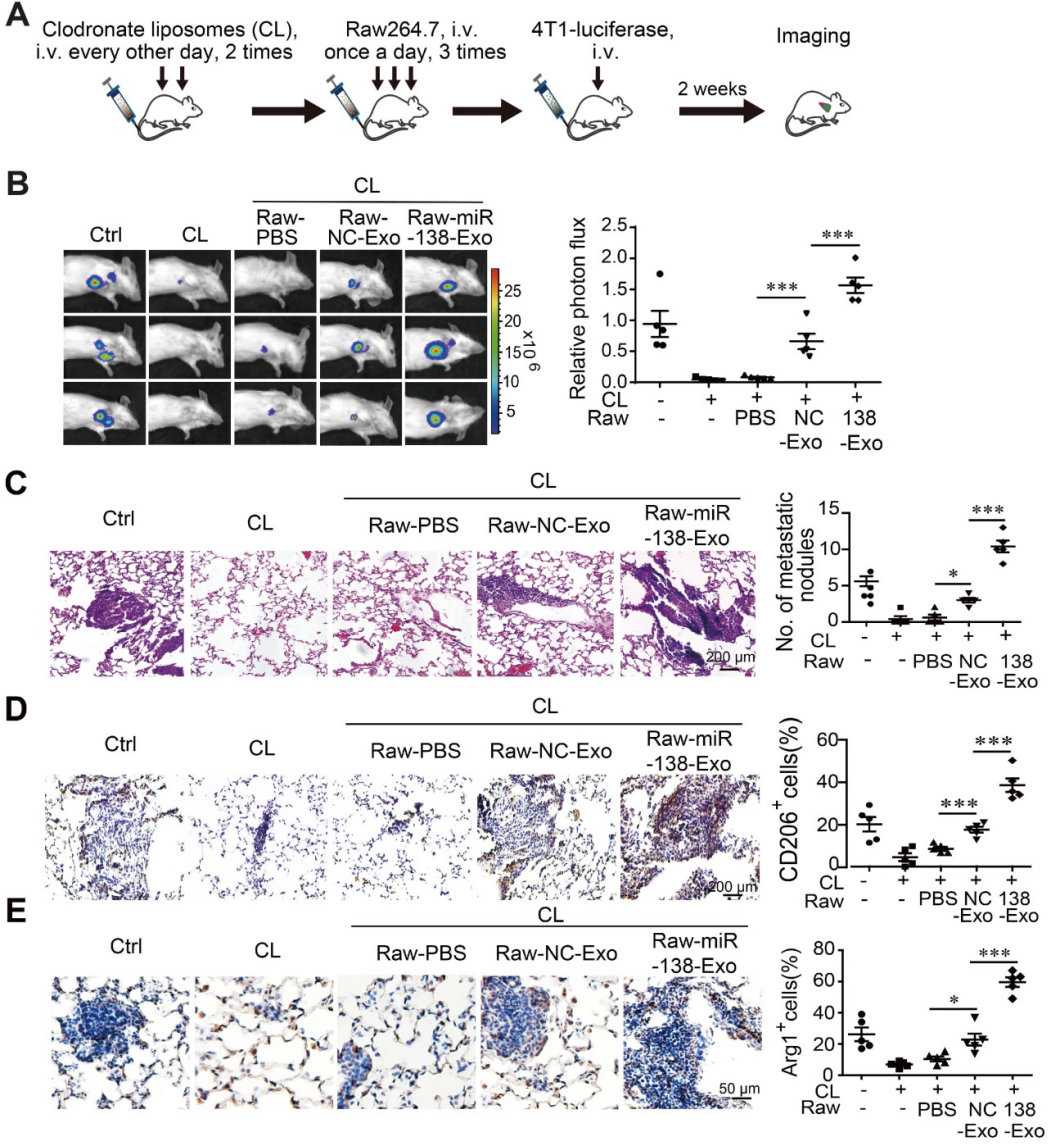
** Exosomal miR-138-5p promotes lung metastasis of breast cancer.** Macrophages were depleted using clodronate liposomes (CL). The mice were then intravenously injected with Raw264.7 cells treated with exosomes followed by tail-vein injection of 4T1-luciferase cells. (A) Schematic of the lung metastasis assay. (B) Representative images of lung luminescence of mice (left) and the analysis of the results of luminescent photon flux (right). (C) Representative images of HE-stained lung tissues (right panel). (D) Representative images of immunohistochemical analysis of CD206 expression in lung tissues (left) and analysis (right). (E) Representative images of immunohistochemical analysis of Arg1 expression in lung tissues (left) and analysis (right). Data are shown as the mean ± SEM. *P < 0.05; **P < 0.01; ***P < 0.001.

Polarized macrophages play an important role in determining the phenotypes of tumor cells. We therefore asked whether M2 macrophages treated with exosomal miR-138-5p contributed to tumor metastasis in a mouse xenograph model of breast cancer. We depleted macrophages from mice using clodronate followed by transfer of Raw264.7 cells treated with exosomes derived from T47D or T47D cells overexpressing miR-138-5p. The mouse model was established through intravenous injection of 4T1-luciferase cells via tail vein (Figure 6A). Metastasis was monitored using an *In vivo* Imaging System Facility (IVIS) system and counting metastatic nodules in the lung.

We found that the incidence of lung metastasis was significantly higher in mice engrafted with macrophages treated with T47D-miR-138-5p exosomes compared with control exosomes (Figure 6B-C). When we determined the percentage of CD206- and Arg1-positive M2 macrophages in tumor tissue, we found that administration of macrophages treated with T47D-miR-138-5p exosomes increased the percentage of M2 cells (Figure 6D-E). Together, these results suggest that M2 macrophages induced by exosomal miR-138-5p promoted the lung metastasis of breast cancer.

To evaluate the potential of miR-138-5p for clinical application, we determined whether its levels correlated with tumor progression in patients with breast cancer. Analysis of TCGA data showed that the levels of miR-138-5p in tumor tissues of breast cancer increased compared with those of normal tissues (Figure S4A). We isolated miR-138-5p from exosomes in sera of patients with benign tumors or different stages of breast cancer. The global level of exosomal miR-138-5p positively correlated with tumors diagnosed greater than stage 0/I, although no significant difference was observed between stage 0/I and benign tumors (Figure S4B). Furthermore, the expression of *KDM6B* was significantly lower in patients with breast cancer compared with normal controls (TCGA dataset) (Figure S4C). The above results indicate that circulating miR-138-5p positively associated with the progression of breast cancer, suggesting its potential role as the prognostic marker for tumor progression.

## Discussion

Macrophages in the tumor microenvironment help maintain the growth and metastasis of tumor cells via reprogramming their phenotypes 3, 29. The highly phenotypic plasticity of macrophages indicates that reprogramming TAMs will serve as an effective approach for designing cancer therapy strategies. Epigenetic regulation is required for the differentiation and functional programming of macrophages 6, 30, leading us to investigate the role of the H3K27 histone demethylase KDM6B in the progression of breast cancer.

Here, we demonstrate that the expression of *KDM6B* was downregulated in macrophages upon coculture of MDA-MB-231 cells. Moreover, THP-1 cells underwent M2-like activation in such cocultures, which was abolished via overexpression of KDM6B. We further found that miRNA-138-5p mediated the inhibition of *KDM6B* expression and that the transfer of exosomal miRNA-138-5p, produced by human breast cancer cells, to macrophages inhibited the expression of *KDM6B*.

The levels of KDM6B increase and then decrease in IL-4 stimulated macrophages, and inhibition of *KDM6B* expression suppresses LPS-induced proinflammatory activation of human primary macrophages 7, 31. Consistent with these findings, in this study we observed a transient increase in KDM6B levels followed by a persistent decrease in macrophages cocultured with MDA-MB-231 cells. Furthermore, overexpression of *KDM6B* promoted and inhibited M1- and M2-polarization, respectively.

KDM6B controls gene expression in LPS-activated macrophages, depending on the level of H3K27me3 associated with KDM6B-target genes 10. In IL-4 stimulated macrophages, KDM6B-mediated H3K27me3 demethylation increases the expression of M2 marker genes 32, and KDM6B is required for M2 polarization during helminth infection 12. Here we show that downregulation of KDM6B expression inhibited the recruitment of KDM6B to the promoters of M1-related genes. Together, these data suggest that KDM6B contributes to the regulation of the polarization of TAMs. However, we are unable to exclude the possibility that other epigenetic modulators were involved.

Evidence indicates that exosomes mediate intercellular communication as delivery cargos, transferring signaling molecules to target cells 25, 33. Among them, miRNAs delivered via exosomes mediate the crosstalk between tumor cells and macrophages 34. Several exosomal miRNAs are associated with an M2-like phenotype. In pancreatic cancer, hypoxia-induced exosomal miR-301 promotes M2-macrophage polarization 27. Moreover, the infiltration of TAMs, influenced by miR-28-5p and IL-34 expression, promotes the metastasis of hepatocellular carcinoma 28.

Exosomal miR-145 expressed by colorectal cancer cells regulates the polarization of TAMs through targeting HDAC11 21. Furthermore, exosomal miR-1246 produced by mutp53 tumor cells reprograms macrophages to promote cancer progression 35. Here, we show that miR-138-5p produced by breast cancer cells was transferred to macrophages via exosomes, resulting in the inhibition of *KDM6B* expression and activity. *In vivo*, in order to define the role of miR-138-5p in the regulation of macrophage phenotype, we added the control Raw 264.7 cells or exosome-treated Raw 264.7 cells to the mice with prior depletion of macrophages 36. Although administration of Raw 264.7 failed to completely rescue the effect of macrophage depletion, exosome-treated Raw 264.7 cells exhibited stronger pro-metastatic effect that the control Raw 264.7 cells in the lung metastatic tumor model. These studies suggest that exosomal miR-138-5p derived from tumor cells influences the reprogramming of macrophages via an epigenetic factor.

The potential utility of circulating exosomal miRNAs as diagnostic markers of tumor progression has been the focus of an increasing number of studies. For example, the levels of miR-1247-3p in serum exosomes of patients with primary hepatic cancer are associated with the extent of lung metastasis 16; and the levels of miR-101 in patients with metastatic osteosarcoma are significantly lower compared with those of healthy controls or patients with nonmetastatic cancers. Extracellular vesicle-miR-101 represents a promising circulating biomarker for metastasis of osteosarcoma 17. Consistent with these findings, in this study we found that the levels of exosomal miR-138-5p in the sera of patients with breast cancer positively correlated with the malignant phenotype, suggesting that miR-138-5p present in circulating exosomes will serve as a prognostic marker of breast cancer. Meanwhile, miR-138-5p may serve as a potential target for the treatment of breast cancer.

In summary, we demonstrate here that miR-138-5p inhibited the activity of the epigenetic factor KDM6B. Cancer cell-derived miR-138-5p transferred to macrophages was involved in regulating the phenotypic programming of macrophages. This effect was associated with the levels of H3K27me3 on M1-associated transcriptional promoters. Exosomes participated in the delivery of miR-138-5p between cancer cells and macrophages, and circulating exosomal miR-138-5p may therefore serve to develop a prognostic index for breast cancer. Our present findings will serve as a platform to develop more effective diagnostics and to identify targets of therapy for managing patients with breast cancer.

## Methods

### Cell culture and reagents

The human breast carcinoma cell lines MDA-MB-231 and T47D were obtained from the American Type Culture Collection (Manassas, VA) and cultured in high-glucose Dulbecco's modified Eagle's medium (DMEM; Biological Industries, Kibbutz Beit-Haemek, Israel) containing 10% FBS (Biological Industries). The murine breast carcinoma cell line 4T1, the human monocyte THP-1 cell line, and the murine macrophage cell line Raw 264.7 were cultured in RPMI-1640 (Biological Industries) containing 10% FBS. Cells were maintained at 37 °C in a humidified atmosphere containing 5% CO_2_. For the coculture assay, MDA-MB-231 and T47D cells (3 × 10^5^) were added to a six-well plate. To prepare cell lysates, THP-1 cells (3 × 10^5^) were added 24 h later to 0.4 μm-diameter culture inserts (Millipore Corp, Billerica, MA) above the cancer cells for 48 h.

### Vector construction and establishment of stable cell lines

Gene expression: The DNA sequence encoding human *KDM6B* was PCR-amplified from the plasmid pCMV-HA-KDM6B (Addgene, Cambridge, MA) and cloned into plasmid pLV-EF1α-MCS-IRES-Bsd (Biosettia, San Diego, CA). Gene silencing: short hairpin RNA (shRNA) sequences targeting *KDM6B* were cloned into the pLV-H1-EF1α-puro vector (Biosettia). Lentiviruses carrying the overexpression vectors, gene silencing vectors, or empty vectors were produced according to the manufacturer's instruction. To establish stable recombinant cell lines, lentivirus-containing medium was added to cells in the presence of 8 μg/mL polybrene for 48 h before selection with 10 μg/mL blasticidin or 1 μg/mL puromycin for 1 week.

### miRNA Transfection

THP-1, MCF7, T47D, or MDA-MB-231 cells were transfected with miRNA mimics or with an miR-138-5p inhibitor using Lipofectamine 2000 Transfection Reagent (Invitrogen) according to the manufacturer's instructions. Negative control (NC) miRNAs, miR-146a mimics, miR-138-5p mimics, and miR-138-5p inhibitors were synthesized by RiboBio (Guangzhou, China).

### RNA isolation and RT-qPCR

Total RNA was extracted using TRIZOL (Invitrogen), and reverse transcription was performed using the TransScript First-Strand cDNA Synthesis SuperMix Kit (TransGen Biotech, Beijing, China) according to manufacturer's recommendations. The primers were designed and synthesized as follows: hsa-miR-138-5p, 5'gtcgtatccagtgcagggtccgaggtattcgcactggatacgaccggcct3'; hsa-miR-146a, 5'gtcgtatccgtgcagggtccgaggtattcgcactggatacgacaaccca3'; hsa-miR-16-5p, 5'gtcgtatccagtgcagggtccgaggtattcgcactggatacgaccgccaa3'; hsa-U6, 5'aaaatatggaacgcttcacgaatttg3'. qPCR was performed using a Real-Time Thermal cycler (Bio-Rad, Hercules, CA) with TransStart Top Green qPCR SuperMix kit (TransGen Biotech). Forward primers were designed using sequences of the mature forms of human miRNAs. The forward primer sequences were as follows: miR-138-5p, 5'cgagctggtgttgtgaatc3'; miR-146a, 5'cgcgtgagaactgaattcca3'; miR-16, 5'cgtagcagcacgtaaata3'. The universal reverse primer was 5'gtgcagggtccgaggt3'. Other primers were as follows: hsa-pri-miR138, forward: 5'gtgttgtgaatcaggccgac3´ and reverse: 5´cgcctggagaggatgggtat3'; hsa-pre-miR138, forward: 5'gtgttgtgaatcaggccgac3' and reverse: 5'tgatgcaaccctggtgtcgt3'; hsa-U6, forward: 5'ctcgcttcggcagcacatatact3' and reverse: 5'acgcttcacgaatttgcgtgtc3'; homo-KDM6B, forward: 5'agcaaacgggatgccttctca3' and reverse: 5'tgttcgccactcgcttccaccag3'; homo-CD163, forward: 5'cgagttaacgccagtaagg3' and reverse: 5'gaacatgtcacgccagc3'; homo-ARG1, forward: 5'tgccctttgctgacatccctaat3' and reverse: 5'cttcttgacttctgccacctt3'; homo-IL-10, forward: 5'gagaaccaagacccagacatca3' and reverse: 5'aaggcattcttcacctgct ccac3'; homo-TGF-β, forward: 5'cgtggagctgtaccagaaatac3' and reverse: 5'cacaactccggtgacatcaa3'; homo-VEGFA, forward: 5'ggagggcagaatcatcacg3' and reverse: 5'gatgtccaccagggtctcg3'; homo-IL-6, forward: 5'actcacctcttcagaacga3' and reverse: 5'tggaaggttcaggttgtt3'; homo-IL-1β, forward: 5'ccagctacgaatctccg3' and reverse: 5'cgttatcccatgtgtcg3'; homo-TNF-α, forward: 5'tgacaagcctgtagccc3' and reverse: 5'cccttgaagaggacctgg3'; mouse-β-actin, forward: 5'cagaaggagattactgctctggct3' and reverse: 5'tactcctgcttgctgatccacatc3'.

### Western blotting

Western blotting analysis was performed according to published protocols. Primary antibodies were as follows: KDM6B (ab38113, Abcam, Inc. Cambridge, MA), CD63 (ab193349), Alix (sc-99010, Santa Cruz Biotechnology, Inc., Santa Cruz, CA), CD9 (555370, BD Biosciences, San Jose, CA), CD163 (ab182422), TNF-α (sc-130349) and anti-β-actin (sc-16632), and goat anti-rabbit or anti-mouse antibody (ZAGB-BIO, Beijing, China) secondary antibodies were conjugated to horseradish peroxidase-. Immunocomplexes were detected using an ECL chemiluminescence kit (Millipore).

### Immunohistochemistry

The detailed immunohistochemistry protocol is published 37. Briefly, tissue sections were incubated with an antibody against CD206 (ab64693, Abcam) and Arg1 (ab239731, Abcam), followed by incubation with a biotinylated secondary antibody and then incubation with an avidin-peroxidase complex. Immunocomplexes were detected using diamino-benzidine, and the cells were counterstained with hematoxylin. Images were acquired using an IX51 Research Microscope (Olympus Co., Tokyo, Japan).

### Isolation of exosomes

Cells were grown in culture medium containing fresh basic medium supplemented with serum, which was depleted of exosomes by ultra-centrifugation at 100,000 × *g* at 4 °C for 16 h. Exosomes were collected from the supernatant after sequential differential centrifugation at 1000 × *g* for 5 min, 2000 × *g* for 20 min, and 5000 × *g* for 30 min, and then at 10000 × *g* for 30 min and 100000 × *g* for 70 min. Exosomes were resuspended in PBS and centrifuged at 100000 × *g* for 70 min.

Exosomes were isolated from the sera of patients with benign breast tumors (n = 15) or breast cancers (n = 39). Exosome Isolation Reagent for plasma or serum (RiboBio) was used to isolate exosomes according to the manufacturer's protocol.

### Flow cytometry

Cells were collected and washed with 1 × PBS containing 1% FBS and incubated with PE-conjugated anti-human CD163 (FAB167P, R&D, Minneapolis, MN) antibody for 15 min in the dark at 4 °C. Cells were analyzed using a FACS Calibur flow cytometer (BD Biosciences, San Jose, CA), and data analysis was performed using Flowjo software (Tree Star, Inc., Ashland, OR).

### Dual-luciferase reporter assay

Potential miR-138-5p binding sites in the *KDM6B* 3´-UTR were predicted using TargetScan7.1 (http://www.targetscan.org). A sequence containing the presumed miR-138-5p binding site of the *KDM6B* 3'-UTR was inserted into the reporter plasmid pmirGLO (Promega, Madison, WI). To determine binding specificity, the seed region of the *KDM6B* 3'-UTR was mutated from CACCAGC to GTGGTCG. THP-1 cells were co-transfected with wild-type or mutated pmirGLO-*KDM6B* 3´-UTR vectors, miR-138-5p mimics, or scrambled-sequence control. Luciferase activity was measured 40 h later using a Dual-Luciferase Reporter Assay System (Promega) according to the manufacturer's protocol. Luciferase activity was normalized to that of Renilla luciferase. To detect the promoter activities of M1-associated genes, the promoters of the genes encoding human IL-6, IL-1β, TNF-α (predicted using software) were each cloned into the pGL4.2-basic luciferase reporter vector (Promega). *KDM6B*-overexpressing or silenced THP-1 cells were transfected with the pGL4.2-IL-6/IL-1β/TNF-α promoter plasmid. The analysis was performed according to the method described above.

### Chromatin immunoprecipitation (ChIP) assay

The ChIP assay was performed using an EZ-ChIP kit (Millipore) according to the manufacturer's instructions. Briefly, cells grown in 10-cm dishes were cross-linked with 1% formaldehyde for 10 min at room temperature, and glycine was used to stop the reaction. After sonication and centrifugation, the supernatant was collected and immunoprecipitated with an anti-KDM6B (ab38113, Abcam) or an anti-H3K27me3 (ab6002, Abcam) antibody. An anti-RNA polymerase antibody and anti-rabbit IgG or anti-mouse IgG were used as positive and negative controls, respectively. Real-time PCR was performed to detect the enrichment of KDM6B or H3K27me3 bound to the promoter regions of genes encoding IL-6, IL-1β, and TNF-α. The primers used were as follows: IL-6, CR1-forward: 5'gctcacattacatagacggatcaca3' and reverse: 5'tgggcatttactcaagttttgtttc3'; CR2-forward: 5'gccgtgcctgcgtccgtagttt3' and reverse: 5'cagtgaggactttgctctctgtgcc3'; CR3-forward: 5'tgtcttgccatgctaaaggacgtca3' and reverse: 5'gccgcccgccagccctccct3'. IL-1β, CR1- forward: 5'ggcgtggcggcaggtgcctgtagt3' and reverse: 5'accaaccaagagttatcagtttctc3'; CR2-forward: 5'ctctgccccagccaagaaaggtcaa3' and reverse: 5'tttcccacgttagaagaatgttt3'; CR3-forward: 5'tctctctgtctctctgcctctttgt3' and reverse: 5' ctcatgttactggtctcagcgtctc3'. TNF-α, CR1-forward: 5'gagaggggaataatagaagaacatc3' and reverse: 5'gcccttatccttttgttctcc3'; CR2-forward: 5'cagggctatggaagtcgagtatggg3' and reverse: 5'cctcaggaaaggctgggtgg3'; CR3-forward: 5'ggacatataaaggcagttgttggc3' and reverse: 5'gctgaggaacaagcaccgcctggag3'.

### Animal models

Female BALB/c mice (6-8 weeks of age) were purchased from Vital River Laboratory Animal Technology Co. Ltd (Beijing, China) and maintained in a specific-pathogen-free facility at Nankai University. Macrophages were depleted via tail vein injection twice, once every other day, of 200 μL PBS (control) or clodronate liposome (5 mg/mL, 40337ES10; Yeasen, Shanghai, China) 38. Two days later, mice were intravenously injected daily 3 days with 2 × 10^5^ Raw264.7 cells treated with PBS, or exosomes derived from T47D-NC or T47D-miR-138-5p cells. Subsequently, intravenous experimental lung metastasis mouse model of breast cancer was established via intravenously injection of 2 × 10^5^ 4T1-luciferase cells in the vein tail 39. To examine the foci of metastatic cells, lung tissues were fixed with 4% PFA for 24 h and stained with hematoxylin and eosin. Metastasis was evaluated by counting metastatic foci in five randomly selected fields.

### Statistical analysis

The data were analyzed using GraphPad Prism5 software (GraphPad Software, San Diego, CA). Results are expressed as the mean ± SEM. Statistical significance was evaluated using a two-tailed Student *t* test or two-way analysis of variance, *P<0.05, **P<0.01, and ***P<0.001 indicate a significant difference.

## Supplementary Material

Supplementary figures and tables.

## Abbreviations

KDM6B: Jumonji domain-containing protein D3
TAMs: Tumor-associated macrophages
miRNA: microRNA
Exo: exosomes
NC: Negative control
3´-UTR: 3´-untranslated regions
WT: wild-type
MT: mutant
IN: inhibitor
CM: conditioned medium
FBS: fetal bovine serum
CL: clodronate liposome
ChIP: Chromatin immunoprecipitation
PFA: paraformaldehyde
H&E: hematoxylin and eosin
IHC: immunohistochemistry
