# Supplementary material for: Cancer-derived exosomal miR-138-5p modulates polarization of tumor-associated macrophages through inhibition of KDM6B
Source: Theranostics. 2021 May 3;11(14):6847–59. doi: 10.7150/thno.51864 (PMC8171095; doi:10.7150/thno.51864)
Supplement: Supplementary file 1 — Supplementary figures and tables. [file thnov11p6847s1.pdf]

Supplementary Information

Supplementary Materials and Methods

Cell culture and reagents

Human breast carcinoma cell lines SKBR3, SUM159 and MDA-MB-468 were obtained from the American Type Culture Collection (Manassas, VA) and cultured in high glucose Dulbecco's modified Eagle's medium (DMEM, Biological Industries, Kibbutz Beit-Haemek, Israel) containing 10% FBS (Biological Industries). Human breast epithelial cell line MCF10A was cultured in DMEM/F-12 (Biological Industries) containing 5% horse serum (Biological Industries), 10 μg/mL human insulin, 0.5 μg/mL Hydrocortisone, 100 ng/mL Cholera toxin (Sigma-Aldrich, St. Louis, MO) and 20 ng/mL hEGF (Invitrogen, Carlsbad, CA,).

Transmission electron microscopy

Isolated exosomes were resuspended in PBS and transferred onto a carbon-coated copper grid. After air drying, exosomes were stained with phosphotungstic acid. Images were captured after drying by TEM (JEM-1230; JEOL, Japan).

Nanoparticle tracking analysis

Exosomes were diluted in PBS and then the concentration and the particle size distribution were analyzed using the ZetaView (Particle Metrix, Germany).

Exosomes labeling and uptake assay

T47D cells were transfected with Cy3-miR-138-5p mimics for 6 h and then treated with 10 μg/mL Dio (3,3′-Dioctadecyloxacarbocyanine perchlorate, Sigma) for 15 min before washing with 1 × PBS. Exosomes in conditioned medium were obtained as described above. For uptake of exosomes, THP-1 cells (1×10^6) were treated with the 50 μg/mL Dio-labeled exosomes for 6 h and collected. Fluorescent and phase contrast images were captured.

Transwell assay

Tumor cell migration was examined using a transwell assay. According to the manufacturer’s protocol, a transwell 24-well Boyden chamber (Corning, USA) with an 8.0-μm pore size polycarbonate membrane was used for cell migration (without

Matrigel) assays. Briefly, 5 × 10^4 tumor cells suspended in 200 µL of serum-free medium were seeded in the top chamber, while the bottom chambers contained 500 µL of medium supplemented with 10% FBS. After incubation at 37 °C in a 5% CO_2 for 8h, the migrated cells in the bottom chamber were stained with 0.1% crystal violet.

Supplementary Figures and Figure legends

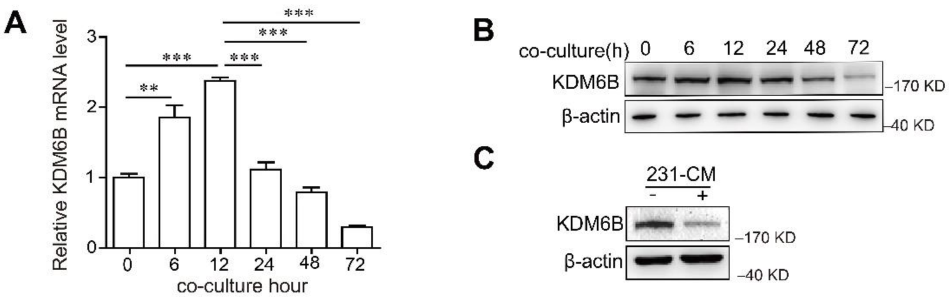

Figure S1. Co-culture with MDA-MB-231 cells suppresses KDM6B in macrophages.
THP-1 cells were co-cultured with MDA-MB-231 cells for 0 h, 6 h, 12 h, 24 h, 48 h and 72 h. **(A)** mRNA level of KDM6B was detected by RT-qPCR in THP-1 cells. **(B)** Western blot analysis of KDM6B expression in THP-1 cells. **(C)** Western blot analysis of KDM6B expression in THP-1 cells treated for 48 h with conditioned medium (CM) derived from MDA-MB-231 cells. Data are shown as the mean ± SEM. **P* < 0.05; ***P* < 0.01; ****P* < 0.001.

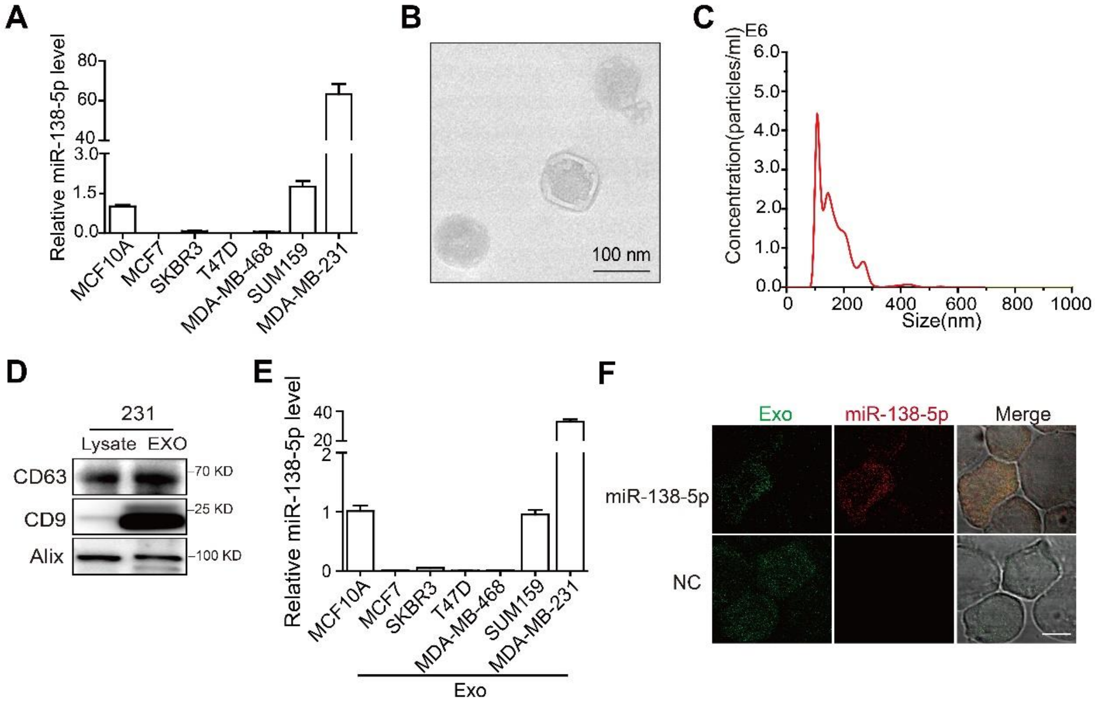

Figure S2. Breast cancer cell-derived exosomes deliver miR-138-5p to macrophages.

(A) RT-qPCR analysis of miR-138-5p in different breast cancer cells. MCF10A serves as a control and data were normalized to levels of U6. Exosomes from MDA-MB-231 cells were isolated by ultracentrifugation. **(B)** Morphological characterization was detected by transmission electron microscopy and **(C)** the size of distribution was measured using nanoparticle tracking analysis. **(D)** western blot analysis of markers of exosomes. **(E)** RT-qPCR analysis of miR-138-5p expression in the exosomes (EV) derived from different breast cancer cells. MCF10A serves as a control and data were normalized to levels of miR-16. **(F)** THP-1 cells were incubated with Dio-labeled exosomes derived from T47D cells transfected with Cy3-miR-138-5p mimics. The representative fluorescent and phase contrast images were shown. Data are shown as the mean ± SEM. *$P < 0.05$; **$P < 0.01$; ***$P < 0.001$.

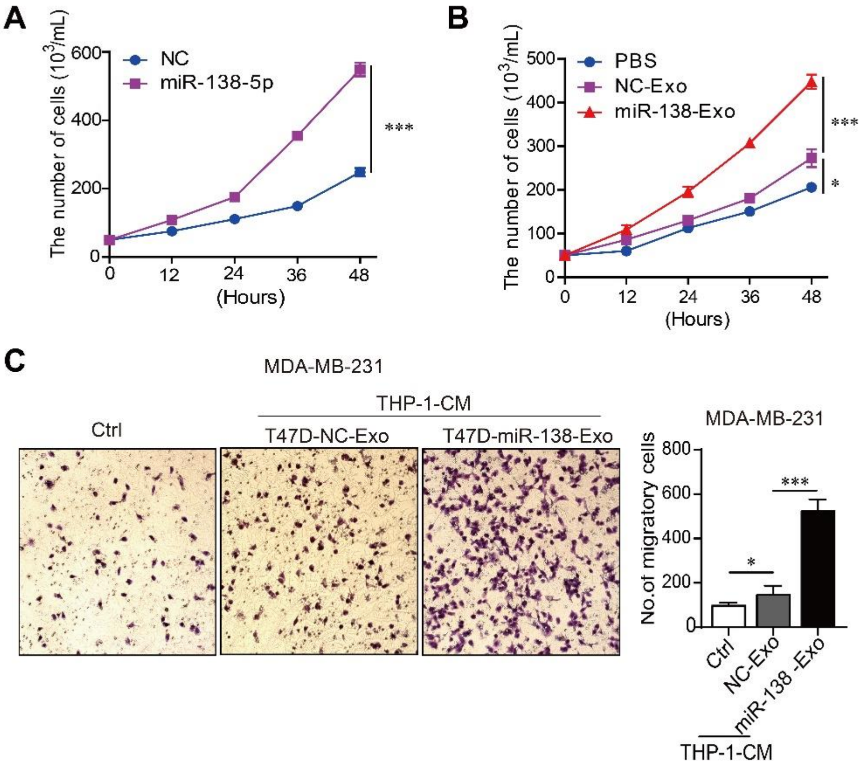

Figure S3. miR-138-5p promotes the M2-like functions of THP-1 cells.

(A) Numbers of THP-1 cells after transfected with negative control (NC) or miR-138-5p mimic at different times. **(B)** Numbers of THP-1 cells treated with exosomes derived from T47D cells transfected with NC or miR-138-5p mimics at different times. **(C)** THP-1 cells were treated with exosome derived from T47D cells transfected with NC or miR-138-5p mimics. Transwell assays to detect the migration of MDA-MB-231 cells treated with conditional medium of THP-1 cells for 48h.

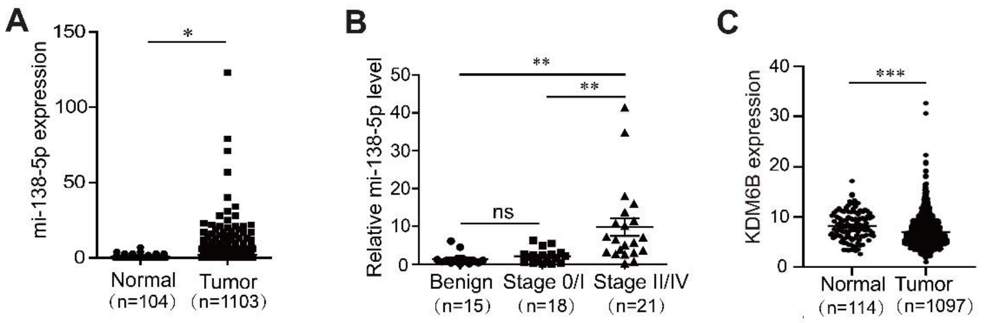

Figure S4. miR-138-5p correlates positively with tumor malignancy in breast cancer patients. **(A)** Comparison of miR-138-5p expression between normal and breast cancer specimens using TCGA database. **(B)** Circulating exosomes were isolated from the serum of benign and stage 0-IV breast cancer patients. The level of miR-138-5p in exosomes was measured by RT-qPCR, normalized to miR-16. **(C)** Analysis of KDM6B levels expressed by normal and breast cancer specimens using TCGA data. Data are shown as the mean ± SEM. $*P < 0.05$; $**P < 0.01$; $***P < 0.001$.
